# An Intelligent Learning System for Unbiased Prediction of Dementia Based on Autoencoder and Adaboost Ensemble Learning

**DOI:** 10.3390/life12071097

**Published:** 2022-07-21

**Authors:** Ashir Javeed, Ana Luiza Dallora, Johan Sanmartin Berglund, Peter Anderberg

**Affiliations:** 1 Aging Research Center, Karolinska Institutet, 171 65 Stockholm, Sweden; ajv@bth.se or; 2 Department of Health, Blekinge Institute of Technology, 371 79 Karlskrona, Sweden; ada@bth.se (A.L.D.); johan.sanmartin.berglund@bth.se (J.S.B.); 3 School of Health Sciences, University of Skövde, 541 28 Skövde, Sweden

**Keywords:** balanced accuracy, bachine learning, oversampling, dementia prediction

## Abstract

Dementia is a neurological condition that primarily affects older adults and there is still no cure or therapy available to cure it. The symptoms of dementia can appear as early as 10 years before the beginning of actual diagnosed dementia. Hence, machine learning (ML) researchers have presented several methods for early detection of dementia based on symptoms. However, these techniques suffer from two major flaws. The first issue is the bias of ML models caused by imbalanced classes in the dataset. Past research did not address this issue well and did not take preventative precautions. Different ML models were developed to illustrate this bias. To alleviate the problem of bias, we deployed a synthetic minority oversampling technique (SMOTE) to balance the training process of the proposed ML model. The second issue is the poor classification accuracy of ML models, which leads to a limited clinical significance. To improve dementia prediction accuracy, we proposed an intelligent learning system that is a hybrid of an autoencoder and adaptive boost model. The autoencoder is used to extract relevant features from the feature space and the Adaboost model is deployed for the classification of dementia by using an extracted subset of features. The hyperparameters of the Adaboost model are fine-tuned using a grid search algorithm. Experimental findings reveal that the suggested learning system outperforms eleven similar systems which were proposed in the literature. Furthermore, it was also observed that the proposed learning system improves the strength of the conventional Adaboost model by 9.8% and reduces its time complexity. Lastly, the proposed learning system achieved classification accuracy of 90.23%, sensitivity of 98.00% and specificity of 96.65%.

## 1. Introduction

Dementia is a mental condition defined by a steady decline in cognitive processes that interfere with everyday living tasks such as memory, problem solving, visual perception, and capacity to focus on a specific task [1]. Usually, older adults are more prone to dementia and many people believe that it is an unavoidable result of aging, which is perhaps incorrect perception. Dementia is not a natural part of the aging process; instead, it should be regarded as a substantial cognitive deterioration that interferes with everyday life. The fundamental cause of dementia development is a variety of disorders and traumas to the human brain [2]. The number of dementia patients is rapidly increasing worldwide and statistical projections suggest that 135 million people might be affected with dementia by 2050 [3]. Furthermore, dementia is ranked on the seventh place in the leading causes of deaths in the world [4] and it is the major cause of disability and dependency among older adults globally [4].

The conventional diagnostic assessment of dementia involves medical history, clinical examinations (e.g., neurological, mental state, and cognitive examinations) and an interview with a relative other than the informant [5]. Current early-stage dementia diagnosis is based on pathological characteristics or cognitive diagnostic tests. Neuroimaging can detect pathology characteristics. Magnetic resonance imaging (MRI) is used to examine the change in neuron-structure [6,7]. The electroencephalography (EEG) is used to evaluate event-related potentials to diagnose early stages of dementia in patients [8,9]. Patel et al., combined EEG and MRI imaging to improve the detection of the early stage of dementia [10]. However, such tools are insufficient for identifying dementia since the cost of testing is prohibitively high, and the testing method is too lengthy and intrusive. Furthermore, recent research suggests computed tomography (CT) or MRI of the brain to rule out structural causes for the clinical phenotype [1,11]. It has been estimated that primary-care clinicians fail to diagnose anywhere from 29% to 76% of patients with dementia or probable dementia [11].

Along with a reliable diagnostic process, appropriate handling must be simple for dementia patients. There are benefits for employing cognitive tests to determine the early stage of dementia since they are quick and easy to do; nevertheless, it is difficult for paramedics to contact patients and promote the testing because elderly individuals often dread attending hospitals. The only way to do tests is through unskilled relatives, who do not completely comprehend the scales. As a result, test findings are often inaccurate. ML algorithms provide a novel answer to this challenge. Paramedics now have an improved access to patients’ lives because of information technology, and they can detect poor cognitive function at an early stage. Additionally, ML algorithms can provide expert medical knowledge. An automated diagnostic systems based on ML techniques can give a high accuracy and user-friendly method of detecting the early stages of dementia. Based on ML approaches, scientists have developed several automated diagnostics systems for various diseases e.g., heart failure [12,13,14,15,16], Parkinson [17], hepatitis [18] and clinical decision support systems [19].

Ana W. Capuano et al. presented an assessment of dementia risk for older adults based on derivation and validation [20]. In their study, the RADaR (Rapid Risk Assessment of Dementia) discrimination was good for the derivation and external-validation cohorts (AUC of the 3-year prediction = 0.82–0.86), compared to age alone (AUC of the 3-year prediction = 0.73), which is a key risk factor for dementia. The inclusion of genetic information did not improve the discrimination. F. V. Cederwald et al., investigated how the continuing trajectory of cardiovascular risk impacts the likelihood of dementia and memory impairment in the future. For this purpose, they used a Bayesian additive regression tree as a multistate survival analysis method [21]. J. R. Cejudo et al. used the cumulative incidence function and inverse probability weighted Cox proportional hazards regression models with adjustments for demographic and clinical covariates, to investigate whether platelet function is associated with dementia risk in the Framingham Heart Study [22]. Statistical models are useful for determining relationships between variables, but they perform poorly when it comes to predicting outcomes, such as disease prediction. ML models often perform better at predicting results, therefore, researchers are employing ML for disease detection.

### 1.1. Machine Learning for Dementia

Several automated diagnostic systems were proposed in the literature for the early detection of dementia using ML approaches. Dallora et al. [23] investigated predictive factors for the 10-year prediction of dementia based on decision trees (DT) using the Swedish National study on Aging and Care (SNAC) database. In their proposed method, they deployed a recursive feature elimination (RFE) feature selection method in order to select the most important variables from dataset for the classification of dementia. Their proposed method based on RFE and DT had achieved the highest area under the curve (AUC) of 74.50%. D. Stamate et al., developed a framework for the prediction of mild cognitive impairment (MCI) and dementia. Their proposed framework was based on the Relief approach paired with statistical permutation tests for feature selection, model training, tweaking and testing using ML algorithms such as random forest (RF), support vector machine (SVM), gaussian processes, stochastic gradient boosting and extreme gradient boosting. The stability of model performances were studied using computationally expensive monte carlo simulations. Their results for the dementia detection were: an accuracy of 88.00%, sensitivity of 93.00%, and the specificity of 94.00%, whereas the results for the moderate cognitive impairment detection showed a sensitivity of 86.00% and specificity of 90% [24]. Visser et al., developed a system for detecting subtypes of dementia from blood samples while utilizing deep learning (DL) and other supervised ML approaches such as RF and extreme gradient boosting. The AUC for proposed DL method was 85% (0.80–0.89), for xgboost it was 88% (0.86–0.89), and for RF it was 85% (0.83–0.87). In comparison, cerebrospinal fluid (CSF) measurements of amyloid, p-tau, and t-tau (together with age and gender) gave AUC values of 78%, 83%, and 87%, respectively by using xgboost [25]. P. Gurevich et al., used SVM and neuropsychologic factors and achieved 89.00% accuracy through their proposed method [26]. M. Karaglani et al., proposed an automated diagnosis system for Alzheimer’s disease (AD) by using blood-based biosignatures. In their proposed method, they employed mRNA-based statistically equivalent signatures for feature ranking and a RF model for classification. Their proposed automated diagnostics system reported an accuracy of 84.60% [27]. E. Ryzhikova et al., analyzed CSF using ML algorithms for the diagnosis of AD. For the classification purpose, artificial neural networks (ANN) and SVM discriminant analysis (SVM-DA) methods were applied for distinguishing AD and hippocampal (HC) participants with 84.00% sensitivity and specificity. The proposed classification models had a high discriminative power, implying that the technique had a lot of potential for AD diagnosis [28]. P.C Cho & W.H Chen designed a double layer dementia diagnosis system based on ML where fuzzy cognitive maps (FCMs) and probability neural networks (PNNs) were used to provide the initial diagnosis at the base layer and Bayesian networks (BNs) were used to provide final diagnosis at the top layer. The highest accuracy reported by their proposed system was 83.00% [29]. Multimodal medical signals fusion for smart healthcare systems also important for designing and development of automated diagnostic systems for the prediction of diseases [30].

### 1.2. State-of-the-Art Work

F. A. Salem et al., presented a regression-based ML model for the prediction of dementia. In their proposed method, they investigated ML approaches for imbalanced classes in the dataset. They started with intentionally oversampling the minority class and undersampling the majority class, in order to reduce the biasness of ML model. Furthermore, they deployed cost-sensitive strategies to penalize the ML models when an instance is misclassified in the minority class. According to their findings, the balanced RF was the most resilient probabilistic model (using only 20 features/variables) with an F1-score of 0.82, G-Mean of 0.88, and AUC of 0.88 using ROC [31]. F.G. Gutierrez et al. had designed an automated diagnostic system for the detection of AD and frontotemporal dementia (FTD) by using feature engineering and genetic algorithms. Their proposed system had obtained an accuracy of 84% [32]. G. Mirzaei & H. Adeli analyzed state-of-the-art ML techniques for the detection and classification of AD [33]. H. Hsiu et al. studied ML algorithms for early identification of cognitive impairment. Their proposed model obtained an accuracy of 70.32% by threefold cross-validation scheme [5]. Several classification models were constructed using different ML models and feature selection methodologies to automate MCI detection based on gait biomarkers. The ML model by A. Shahzad et al. [34] used for mild cognitive impairment (MCI) pre-screening based on inertial sensor-derived gait biomarkers achieved an accuracy of 71.67 % and sensitivity of 83.33 %.

ML algorithms work best when the samples are roughly evenly split in the dataset. However, dementia has a rather uncommon occurrence, thus balancing sampling the sample must occur in order to build datasets.

### 1.3. Aim of Study

In this article, we have addressed two challenges of dementia prediction using the SNAC dataset, such as bias in the developed ML models and lower accuracy of dementia detection. To show the problem of bias in ML models, we have constructed and trained six distinct ML models i.e., Logistic Regression (LR), K Nearest Neighbors (KNN), Gaussian Naive Bayes (GNB), Support Vector Machine (SVM), Decision Tree (DT), and Random Forest (RF). We used the synthetic minority oversampling technique (SMOTE) method to overcome this problem. The second issue is a poor rate of accuracy for dementia prediction while using SNAC dataset. We develop an intelligent learning system that is a hybrid with autoencoder and adaptive boosting (Adaboost) learning models to address the issue of low accuracy of dementia detection. The autoencoder is utilized for feature extraction, whereas Adaboost is employed for the classification of dementia patients versus healthy subjects. The experimental findings clearly reveal that the offered solutions assist in the alleviation of both problems to some extent.

It is important to note that dementia has numerous subtypes with the most prevalent being Alzheimer’s disease, Vascular dementia, dementia with Lewy Bodies, and Frontotemporal dementia. However, mixed pathologies are not uncommon, particularly Alzheimer’s disease often coexists with Vascular or Lewy Bodies dementia. In addition, unusual subtypes are sometimes mistakenly diagnosed for Alzheimer’s disease [35]. The research described here makes no difference between subtypes, and the word “dementia” refers to all types of dementia.

## 2. Materials and Methods

### 2.1. Dataset Description

The data utilized in this study is a subset of the Swedish National Study on Aging and Care (SNAC). The SNAC is a longitudinal cohort that has been collecting multifactorial data from the Swedish older adult population with the goal of “creating trustworthy, comparable, longitudinal datasets” that will represent an effective infrastructure for aging research and care provision to the elderly [36]. The SNAC (https://www.snac-k.se/) was created as a multipurpose project to study the health and social care of the aging population, and it includes a database consisting of records about physical examination, psychological assessment, social factors, lifestyle factors, medical history etc.

The SNAC data is gathered from four different locations, which represent two Swedish counties i.e., borough and municipality: Skåne, Blekinge, Kungsholmen, and Nordanstig. The SNAC-Blekinge baseline assessment is selected in this study, with data collected from 2000 to 2003. Although there is evidence in the literature that environmental variables may have a role in the incidence of dementia [37,38], this study is based on generic criteria and no distinctions are made between urban and rural locations. Subjects are excluded from this study based on the following criteria: (i) subjects who already had dementia at baseline; (ii) subjects who have missing values at the outcome variable (dementia diagnosis at the 10-year mark); (iii) subjects who have more than 10% missing values in the input variables; (iv) subjects who died before the 10-year study mark; and (v) subjects who were diagnosed with dementia before the 10-year mark, as they could already have advanced dementia.

The SNAC Blekinge baseline included 1402 people. Following the application of aforementioned exclusion criteria, the research sample consisted of 726 people (313 males and 413 females), of which 91 (12.5 %) had dementia at the 10-year point and 635 (87.5 %) did not. Table 1 shows the demographics of research sample in the selected dataset. The variables selection from the SNAC-Blekinge database was based on information from the literature that indicate the impact of selected variables on the dementia disorder [39,40]. It is noteworthy during the selection of variables from SNAC-Blekinge database that there were no differences established between dementia subtypes since mixed pathologies are widespread and rare subtypes are frequently misdiagnosed as Alzheimer’s disease [35].

It is also worth mentioning that all of the variables used for the SNAC project were chosen based on evidence of importance in the aging process (health/disease, social and support network, lifestyle factors, material conditions, and personal resources), as well as statistics on care service utilization [36]. At the study’s baseline (2000–2003) 75 variables were chosen from the following categories: demographic, social, lifestyle, medical history, blood test, physical examination, psychological, and the assessment of numerous health instruments related to dementia evaluation. The list of selected variables can be depicted from Table 2.

The target variable that is used to predict the dementia by the proposed model is given by medical doctors at the mark of 10 years following the SNAC baseline. The International Statistical Classification of Diseases and Related Health Problems-10th Revision (ICD-10) and the Diagnostic and Statistical Manual of Mental Disorders (DSM-IV) were used to make the dementia diagnosis (DSM-5).

### 2.2. Data Preparation

To deal with missing data, the K-Nearest Neighbors (KNN) multiple imputation method was used [51]. This strategy works by locating the K data entries which are most similar (near) to a missing data item. The KNN imputation fills in the missing values with the mean (for numeric variables) or the most common value (for categorical variables) of the K, the most similar neighbors [51]. In this study, the KNN imputation was used independently on items from the majority (no dementia at 10 years) and minority classes (dementia at 10 years mark) because of the significant class imbalance (12.5% on the minority class against 87.5% on the majority class). This way the danger of contaminating the minority class with data from the majority class was reduced. This is consistent with the literature on missing values on binary answer decision trees, which has demonstrated that when imputation is done independently, classification performance improves [52]. After dealing with missing values in the dataset, we have performed a normalization and standardization operation on the selected dataset to improve the quality of data [53]. The performance of ML algorithms improves after data standardization.

Since the dementia dataset has only numeric values with different scales, we have applied a standard-scaler function on the data. The standard-scalar function work as rescaling the distribution of the data values so that the mean of observed value is 0 and standard deviation is 1 [54].

### 2.3. Proposed Model

**Figure 1:**
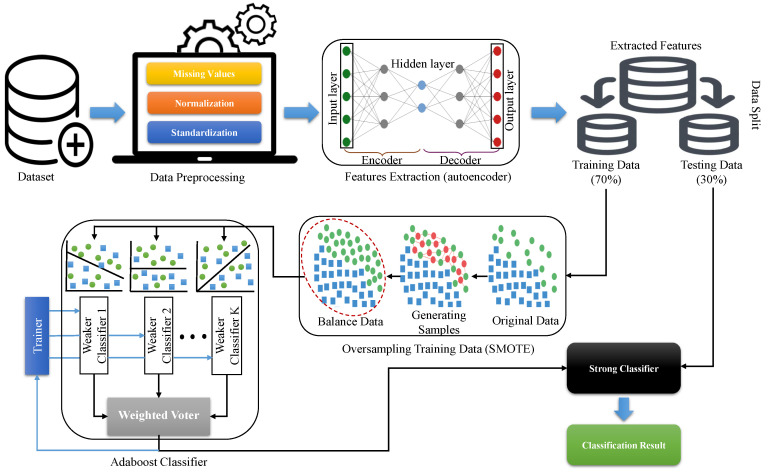
Schematic overview of the proposed intelligent learning system.

In this paper, we developed an intelligent learning system for dementia detection using electronic health record (EHR) data. The proposed learning system is cascaded by three algorithms i.e., autoencoder with synthetic minority oversampling technique (SMOTE) and Adaboost ensemble learning model in order to improve the performance of the proposed model. The autoencoder is used for features extraction so that the proposed model does not learn noisy or irrelevant information from the feature space which causes overfitting in the ML model. SMOTE is deployed to deal with the problem of imbalance classes in the dataset and Adaboost model is used as a predictive model to detect presence or absence of dementia in the population of older adults. To understand the working of proposed learning system, Figure 1 presents an overview of the newly developed model.

Hereby, the operation of proposed learning system components, namely autoencoder, SMOTE, and Adaboost, are given as follows:

The architecture of the autoencoder consists of two parts, one is the encoder while other is the decoder. The encoder part is used to extract features from the reduced dataset. The feedforward neural network is the simplest form of autoencoder with a single layer perceptron that participates in a multilayer perceptron (MLP) with one or more hidden layers between the input and output layer of the neural network. The number of neurons in input and output layer are equal. The purpose behind the equal number of neurons is to reduce the information loss from the original dataset. Thus, autoencoder uses unsupervised learning. The mathematical formation of autoencoder and decoder is given as:

(1) $$\sigma :\lambda \to \omega ,\;\partial :\omega \to \lambda :\;\sigma ,\partial =arg_{\sigma ,\partial }min{∥\lambda -(\partial \circ \sigma )\lambda ∥}^{2}$$

where σ and *∂* are the transition of autoencoder and λ is a given input to target value ω. The hidden layer of neural network take input at the encoding stage is given as:

(2) $$X\;\varepsilon \;{℧}^{t}=\lambda \;and\;map\;to\;h\;\varepsilon \;{℧}^{n}=\omega$$

(3) h=Υ(βX+u)

where *h* is the data and referred as code, latent variables σ is an activation function, such as sigmoid function. β is a weight matrix and *u* is a bias vector. Weight and biases values are iteratively updated by backpropagation during training phase.

The decoder stage of the autoencoder maps $h^{\prime }$ to the features extraction $X^{\prime }$ of the same shape of *X*.

(4) $$h^{\prime }=\Upsilon^{\prime }\left(\beta^{\prime }X+u^{\prime }\right)$$

where $\Upsilon^{\prime }$, $\beta^{\prime }$ and $u^{\prime }$ might be irrelevant to corresponding Υ, β and *u* for the encoder.

For minimize the reconstruction errors of the autoencoders during training phase is referred as information loss and given as:

(5) $$\Psi \left(X,X^{\prime }\right)={∥X-X^{\prime }∥}^{2}={∥x-\Upsilon^{\prime }\left(\beta^{\prime }\left(\Upsilon \left(\beta X+u\right)\right)+u^{\prime }\right)∥}^{2}$$

where, *X* is the average on the training set and autoencoder training is performed through backpropagation of the error, such as other feedforward neural networks.

**Figure 2:**
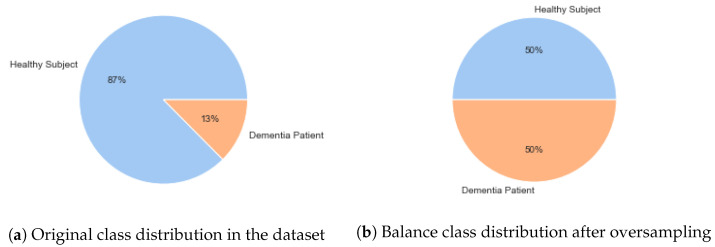
Class distribution before and After applying SMOTE.

Following the features extraction from autoencoder, data partitioning occurs for training and testing of the proposed predictive model. The SMOTE was employed to prevent biasness in proposed learning model for an unbiased prediction of dementia [55]. SMOTE generates synthetic samples of the minority class that results in balanced classes in the dataset. Thus, proposed learning system is trained on balance data by avoiding the biasness factors of ML model due to imbalance classes in the dataset. It is important to mention that the SMOTE is applied on training data following data partitioning. If the SMOTE algorithm is used for balancing the classes on the whole dataset (i.e., prior to data partitioning) then, it would result in biased performance of ML model since samples from the testing dataset would also be included in the training dataset [56]. SMOTE, in contrast to other oversampling approaches, acts in the feature space rather than the data space [55]. It synthesizes (i.e., oversamples) minority class samples by taking a sample from the minority class and creating new samples along the line that links any or all of the k-minority class nearest neighbors. Figure 2 presents the minority and majority class distribution in the dataset before and after the implementation of SMOTE. In this study, we use “imbalanced learn”, a Python-based library to employ the SMOTE technique [57].

Boosting is an ensemble learning strategy that combines the learning ability of weak learners to construct a strong learning model. Freund and Schapire introduced the first practical boosting ensemble model, adaptive boosting or Adaboost [58]. In other words, the Adaboost model transforms a collection of weak classifiers or estimators into a powerful one. It combines the result of various learning algorithms (weak learners or estimators) by assessing their weighted total, which represents the boosting ensemble model’s ultimate output. The final equation of the Adaboost model for classification is as follows:

(6) $$U\left(x\right)=sign\left(\sum_{t=1}^{T}\lambda_{t}u_{t}\left(x\right)\right)$$

where $\mu_{t}$ represents the *t^th^* weak classifier and $\lambda_{t}$ is its associated weight. Equation (6) shows that the Adaboost model is a weighted mixture of *T* weak learners or estimators. Details on the operation and formulation of the Adaboost model can be found in [59,60]. In this study, we briefly explore the Adaboost model formulation as follows:

For a given dataset with n occurrences and binary labels (i.e., taking the case of binary classification as studied in this research), the feature vector *v* and class label *c* may be represented as $v_{i}$ε$R^{h}$, $c_{i}$ε$\left\{-1,+1\right\}$ where −1 represents the negative class (absence of dementia) and +1 represents the positive class(presence of dementia). Weights for each data point are initialized in the first phase as follows:

(7) $$\varpi \left(v_{i},c_{i}\right)=\frac{1}{n}\;\;\;\;\;\;\;i=1,2,3,...n$$

Then, we iterate from *t* = 1 to *T*, applying weak classifiers to the dataset and selecting the one with the lowest weighted classification error.

(8) $$k_{t}=K_{\varpi t}\left[1_{c\neq u(x)}\right]$$

The weight for $t^{th}$ weak classifier or estimator is then determined as follows:

(9) $$\gamma_{t}=\frac{1}{2}ln\left(\frac{1-k_{t}}{k_{t}}\right)$$

Any classifier (weak estimator) with an accuracy greater than 50% will have a positive weight. Furthermore, larger weights will be assigned to more accurate classifiers. Classifiers with less than 50% accuracy, on the other hand, will have negative weights. Adaboost combines such classifier predictions by flipping their sign. As a result of the sign flipping of its prediction, a classifier with 30% accuracy can be changed into one with 70% accuracy. Only classifiers with an exact 50% accuracy have no contribution to the final prediction.

(10) $$\varpi_{t+1}\left(v_{i},c_{i}\right)=\frac{\varpi_{t}\left(v_{i},c_{i}\right)exp\left[-\gamma_{t}c_{i}u_{t}\left(x_{i}\right)\right]}{Z_{t}}$$

where $Z_{t}$ is a normalizing factor used to make the sum of all instance weights equal to one. Additionally, it is evident from Equation (10), that the “exp” term will always be greater than 1 when the misclassified example is from a positive weighted classifier (i.e., $\gamma_{t}$ is always positive and *c × u* is always negative). After each cycle, the misclassified instances will be updated with higher weights. The same concept is used for negative weighted classifiers, with the exception that the initial accurate classifications become misclassifications once the sign is flipped. Finally, after *T* iterations, the Adaboost model will acquire a final prediction by averaging each classifier’s weighted prediction (i.e., weak estimator).

In this research work, we implemented the Adaboost ensemble model in Python software package using scikit-learn module [61]. The Adaboost model’s hyperparameter, i.e., the number of estimators used to generate the final ensemble model, will be indicated by $N_{est}$. Furthermore, the decision tree classifier is employed as the basis estimator. To improve classification performance, we use an exhaustive search technique to find the ideal value of hyperparameter of Adaboost model (i.e., $N_{est}$, learning rate: $l_{r}$) that results as the optimal Adaboost model which helped to yield best performance.

### 2.4. Validation & Evaluation

To test the efficacy of the proposed learning system, we employ holdout validation scheme and cross-validation. For hold-out validation, we split the dataset into 70% and 30% ratio for training and testing purposes, respectively. To establish the efficacy of the proposed learning system, we tested the proposed model against a range of evaluation metrics. i.e., accuracy, sensitivity, specificity, F-score or F-measure, and Mathew’s correlation coefficient (*MCC*). To test the efficacy of the proposed learning system using receiver operator curve (ROC) and area under the curve (AUC), we employ a stratified k-fold validation strategy with k = 6. Traditional accuracy metrics fail to reflect a model’s genuine behavior, as illustrated in experiment 1 of Section 3 of this study. Thus, we used the balanced accuracy metric, which more accurately reflects the real behavior of the built models [62,63,64]. Pereira et al. [65] utilized a similar accuracy metric (global accuracy [65]) that was proposed by Papa et al. [66]. This accuracy metric is also a suitable choice for reflecting a model’s genuine behavior when trained on imbalanced data. In the following formulation, *ACC* stands for the commonly used accuracy metric, while $ACC_{bal}$ stands for the balanced accuracy metric. The mathematical formulation of the used assessment metrics is given as follows:

(11) $$Accuracy=\frac{TP+TN}{TP+TN+FP+FN}$$

where *TP* stands for the number of true positives, *FP* stands for the number of false positives, *TN* stands for the number of true negatives, and *FN* stands for the number of false negatives.

(12) $$Sensitivity=\frac{TP}{TP+FN}$$

(13) $$Specificity=\frac{TN}{TN+FP}$$

(14) $$ACC_{bal}=\frac{Sensitivity+Specificity}{2}$$

(15) $$MCC=\frac{TP\times TN-FP\times FN}{\sqrt{\left(TP+FP)(TP+FN)(TN+FP)(TN+FN\right)}}$$

(16) $$F=\frac{2TP}{TP+FN+FP}$$

In a statistical analysis of binary classification problem, *F* signifies F-score, also known as F-measure or F1 score. F-score yields a value range between 0 and 1, where 1 represents perfect forecasts and 0 represents the poorest. *MCC* is used to assess the correctness of a test. *MCC* can have a value range between −1 and 1, where 1 represents the perfect forecast and −1 represents the poorest forecasts. Consider an example of 100 individuals among them 90 people sufferers from dementia and 10 individuals are healthy subjects, to highlight the benefits of employing a balanced accuracy metric. If we build a model that always predicts a subject to be a dementia patient, then it would have 100% sensitivity but 0% specificity and traditional accuracy of 90%. However, the balance accuracy will be 50%. It is evident that the true behavior of the constructed model is reflected by balanced accuracy, because model can detect only one class but completely failed to detect the second class. However, the traditional accuracy failed to describe the genuine behavior of the constructed model.

## 3. Results

Three different types of experiments were carried out to rigorously assess the performance and efficacy of the newly proposed system for dementia prediction. In the first experiment, we have demonstrated the impact of imbalance classes in the dataset using six conventional ML models. While in the second experiment, the traditional Adaboost algorithm is fine-tuned using a grid search algorithm and tested on the balance dementia dataset. The second experiment is extended and in the second phase of the experiment, the dataset is preprocessed through data standardization and normalization. Following that, the newly proposed method based on autoencoder and Adaboost is tested on the processed data with balanced classes in dataset using the SMOTE method. We have also compared the results of the newly proposed model against the traditional Adaboost model on the balanced dataset. In the third experiment, other conventional ML methods are fine-tuned and tested with features extracted from the autoencoder on the same balanced dementia dataset for performance comparison. All experiments are carried out on a system powered by an Intel (R) Core (TM) i5-8250U CPU running at 1.60GHz and running Windows 10 Home 64bits(Blekinge Institute of Technology, Karlskrona, Sweden) as the operating system. All of the experiments make use of the Python software package as a software tool.

### 3.1. Experiment 1: Impact of Imbalance Classes in the Dataset

In this section, we have employed several ML models (NB, LR, kNN, SVM, RF, DT) to demonstrate the impact of imbalanced data for the prediction of dementia. From the Table 3, it can be depicted that ML models are sensitive to the imbalanced data. The employed ML models are clearly biased in favor of majority class. For instance, it can be observed from the Table 3 that we obtained high a rate of specificity and low rate of sensitivity (see Table 3) when ML models are trained on imbalanced data.

**Figure 3:**
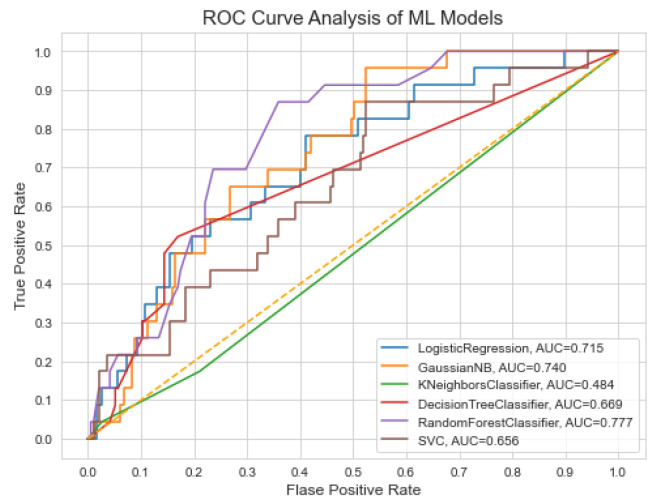
ROC of ML models for dementia prediction.

To avoid this biasness problem, we take a step to balance the training data and for this purpose we deployed the SMOTE method [55] to balance the size of each class in the training data. After balancing the data, it is evident from Table 4 that the performance of ML models is improved i.e., the ML models do not suffer from the biased performance as it can be seen from the values of specificity and sensitivity. Furthermore, we have also studied the performance of ML models based on the ROC evaluation metric while using imbalance data. Figure 3 presents the performance evaluation of ML models based on AUC by using ROC curve.

### 3.2. Experiment 2: Comparative Study with Conventional Adaboost for Dementia Prediction

**Figure 4:**
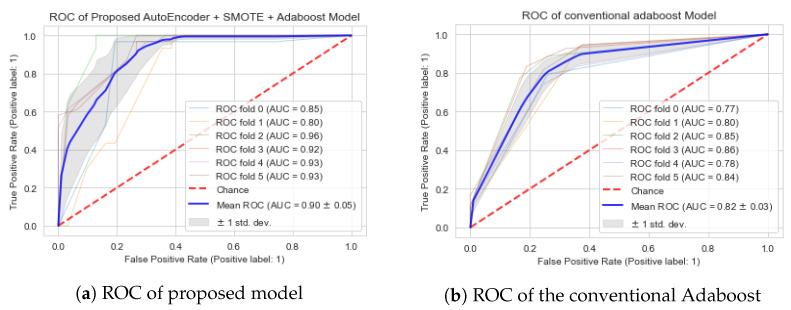
Performance comparison of proposed model with conventional Adaboost model in term of area under the cruve.

This experiment has two phases: in the first phase, we deployed a conventional Adaboost model with hyperparameters fine-tuned using a grid search algorithm on the balanced dataset. The performance of this model is assessed using the ROC. Figure 4b, presents the results of this experiment, in which the conventional Adaboost obtained an average accuracy of 82.00% using all 75 features of the balanced dataset based on the k-fold evaluation metric. While in second phase of this experiment, we evaluated the performance of the newly proposed autoencoder-SMOTE-Adaboost model for dementia patient classification. We employed the autoencoder to extract features from the dataset, which not only helped to increase the Adaboost accuracy but also greatly decreased the time complexity of the proposed model by reducing the data dimensionality. The extracted features by the autoencoder are given as input to the Adaboost model and the hyperparameters of the Adaboost was fine-tuned using a grid search algorithm that assisted in determining the optimal number of estimators (N${}_{est}$) and the learning rate of the Adaboost model. The obtained accuracy along with other performance evaluation metrics are given in Table 5. It can be depicted from Table 5 that the newly proposed autoencoder-SMOTE-Adaboost model has achieved the best accuracy on testing data of 90.23% and an accuracy of 92.10% on training data. This was achieved with the best number of estimators (N${}_{est}$) of 10 and learning rate (l${}_{r}$) of 0.05.

Furthermore, the result of this experiment can be observed from the Figure 4a where the proposed model achieved an average AUC of 90.00% based on the k-fold evaluation metric. For both phases of the experiments, we have taken the same value of K = 6, so that fair comparison is done. The overall performance comparison based on AUC between conventional Adaboost model and the proposed model on balanced dataset is shown in the Figure 4.

### 3.3. Experiment 3: Performance Comparison of the Proposed Model with Other ML Models

We have constructed various comparable prediction systems such as hybridizing autoencoder with Naive Bayes (NB), Logistic Regression (LR), Random Forest (RF), Decision Tree (DT), K Nearest Neighbors (KNN) and Support vector machine (SVM) to test the efficiency of the newly proposed learning system. Table 6 presents the outcomes of each constructed hybrid model. It is noteworthy that all of these constructed hybrid models are tested on balanced data using SMOTE technique for data balancing.

From Table 6 it can be observed that the newly proposed model has achieved the accuracy of 90.23% while using only a small subset of extracted features (06) in comparison to the rest of the ML models. Hence, the proposed model also reduces the complexity of the Adaboost predictive model as training on a smaller number of features will result in reducing training time of the ML model.

## 4. Discussion

In this study, an intelligent learning system is presented for the prediction of dementia using the SNAC dataset. We used 75 features from the SNAC dataset related to demographic, social, lifestyle, medical history, biochemical tests, physical examination, psychological assessment and diverse health instruments relevant to the dementia disorder.

To improve the accuracy of the proposed model along with lower time complexity, we have deployed an autoencoder to reduce the data dimensionality. Based on an artificial neural network, the autoencoder helped to extract useful features from the feature space. After extracting features from the dataset, it was observed that the classes in the dataset were highly imbalanced. To balance the class distribution in the training set of the classifier, we have used SMOTE and for the classification of dementia patients the Adaboost ensemble model was employed. The hyperparameters of the Adaboost model were fine tunned using a grid search algorithm. Thus, the proposed learning model consist of two modules which are hybrid as a single system.

From the results, it can be observed that the proposed model dealt effectively with both problems, imbalance classes in dataset and lower accuracy of ML models for dementia prediction. Experiment 1, addressed the impact of imbalanced classes in the dataset for the prediction of dementia. From Table 3, it can be observed that performance of ML models tends to bias toward the majority class with the ML models achieving higher results of specificity and lower results for sensitivity. This means that the ML models tend to bias the majority class in the dataset. The proposed model has not only resolved the issue of bias results but also improved the accuracy of dementia prediction. Table 5 presents the performance of the proposed model along with hyperparameter values of the Adaboost model. It can be depicted from the Table 5 that the newly proposed model achieved the highest accuracy of 90.23% on testing data, training accuracy of 92.10%, sensitivity of 98.00% and specificity of 96.65% while using only 6 features, which are extracted by the autoencoder. The learning rate of the Adaboost was (l${}_{r}$) 0.05 and the number of estimators were (N${}_{est}$) 10.

Furthermore, we have also compared the results of the newly proposed model with other state-of-the-art ML models which were proposed in the literature for dementia prediction. It can be observed from the Table 7 that the proposed model has achieved significantly improved results when compared to other ML models.

## 5. Conclusions & Future Work

In this paper, we have identified the problems of lower accuracy and bias in the ML models due to imbalanced classes in the dataset for dementia prediction. From experiments, it is demonstrated that when ML models are trained on imbalanced data, their performance is skewed towards the data’s majority class. As a result, for the dementia detection problem, we found a high rate of specificity, but a poor rate of sensitivity since the dementia patient’s class was in the minority and healthy subject class was in the majority. To deal with the bias problem, we presented a novel diagnostic system for the detection of dementia. In our proposed model, the SMOTE technique is employed to eliminate the problem of imbalanced classes in the dataset. The proposed model has two main components which are hybridized and work as a single learning system. The first component work to extract useful features from the dataset for reducing data dimensionality, which helps to lower the computational complexity of the proposed model and improve the accuracy of dementia prediction. For this purpose, we have employed an autoencoder which has reduced the number of features from 75 to 6. The second component of the newly proposed model works as classifier and for this task, we utilized the Adaboost classifier. The hyperparameters of Adaboost model were fine-tuned using a grid search algorithm. From the experimental results, it is observed that the newly proposed model has outperformed the traditional Adaboost model along with other state-of-the-art ML models that also used extracted features from the autoencoder. It was also observed that the proposed learning system improved the performance of a conventional Adaboost model by 9.8%. Moreover, the proposed model has also demonstrated a lower time complexity as compared to the traditional Adaboost model because the proposed model uses a fewer number of features than the traditional Adaboost model.

Therefore, the problem of bias in the developed ML models was avoided in this work, and an unbiased learning model was designed that enhanced dementia diagnosis accuracy while also lowering the complexity of ML models by reducing the number of features. However, the achieved accuracy still needs significant improvement. This is a shortcoming of this study. Future research should focus on developing more robust models that can enhance dementia diagnosis accuracy while keeping the unbiased behavior of the developed models. This could be possible by combining feature extraction approaches with deep learning models. Furthermore, when the number of samples in the dataset is large, the performance of ML techniques improves. The dataset employed in this study has only 721 samples, which is rather small in terms of sample size. As a result, researchers must develop datasets with large sample sizes, particularly for dementia.

## Figures and Tables

**Table 1:** Demographic overview of the samples in the dataset.

Age_Group	Male	Female	Subj.${}_{\mathrm{Sum}}$	Diagnosis.${}_{\mathrm{Dementia}}$
60	82	82	164	02
66	75	95	170	06
72	50	74	124	10
78	41	50	91	17
81	35	46	81	19
84	26	42	68	22
87	04	19	23	14
90+	00	05	05	01
**Total**	313	413	726	91

**Table 2:** Overview of selected variable.

Variable_Category	Variables_Names	Sum
Demographic	Age, Gender	02
Social	Education, Religious Belief, Religious Activities, Voluntary Association, Social Network, Support Network, Loneliness	07
Lifestyle	Light Exercise, Alcohol Consumption, Alcohol Quantity, Work Status, Physical-Workload, Present Smoker, Past Smoker, Number of Cigarettes a Day, Social Activities, Physically Demanding Activities, Leisure Activities	11
Medical History	Number of Medications, Family History of Importance, Myocardial Infarction, Arrhythmia, Heart Failure, Stroke, TIA/RIND, Diabetes Type 1, Diabetes Type 2, Thyroid Disease, Cancer, Epilepsy, Atrial Fibrillation, Cardiovascular Ischemia, Parkinson’s Disease, Depression, Other Psychiatric Diseases, Snoring, Sleep Apnea, Hip Fracture, Head Trauma, Developmental Disabilities, High Blood Pressure	22
Biochemical Test	Hemoglobin Analysis, C-Reactive Protein Analysis	02
Physical Examination	Body Mass Index (BMI), Pain in the last 4 weeks, Heart Rate Sitting, Heart Rate Lying, Blood Pressure on the Right Arm, Hand Strength in Right Arm in a 10s Interval, Hand Strength in Left Arm in a 10s Interval, Feeling of Safety from Rising from a Chair, Assessment of Rising from a Chair, Single-Leg Standing with Right Leg, Single Leg Standing with Left Leg, Dental Prosthesis, Number of Teeth	13
Psychological	Memory Loss, Memory Decline, Memory Decline 2, Abstract Thinking, Personality Change, Sense of Identity	06
Health Instruments	Sense of Coherence [41], Digit Span Test [42], Backwards Digit Span Test [42], Livingston Index [43], EQ5D Test [44], Activities of Daily Living [45], Instrumental Activities of Daily Living [46], Mini-Mental State Examination [47], Clock Drawing Test [48], Mental Composite Score of the SF-12 Health Survey [49], Physical Composite Score of the SF-12 Health Survey [49], Comprehensive Psychopathological Rating Scale [50]	12

**Table 3:** Performance of conventional ML predictive models on imbalanced dataset, Where *Acc${}_{Train}$*: Accuracy on training data, *Acc${}_{Test}$*: Accuracy on test data, Sens: Sensitivity, Spec: Specificity, *MCC*: Matthews correlation coefficient.

Model	*Acc*${}_{\mathrm{Train}}$ (%)	*Acc*${}_{\mathrm{Test}}$ (%)	Sens. (%)	Spec. (%)	F1_Score	*MCC*
NB	82.57	74.10	22.22	91.10	74.00	0.1428
LR	85.32	71.15	23.53	90.55	71.00	0.1228
RF	89.55	76.50	15.36	89.40	77.00	0.2278
DT	71.45	66.50	25.93	91.62	67.00	0.1882
kNN	78.56	48.40	16.67	89.62	49.00	0.0335
SVM	86.69	65.60	31.25	91.09	66.00	0.1896

**Table 4:** Performance of conventional ML predictive models on balanced dataset, Where *Acc${}_{Train}$*: Accuracy on training data, *Acc${}_{Test}$*: Accuracy on test data, Sens: Sensitivity, Spec: Specificity, *MCC*: Matthews correlation coefficient.

Model	*Acc*${}_{\mathrm{Train}}$ (%)	*Acc*${}_{\mathrm{Test}}$ (%)	Sens. (%)	Spec. (%)	F1_Score	*MCC*
NB	75.37	70.70	98.57	78.89	70.00	0.2287
LR	82.74	76.85	85.35	80.55	77.00	0.4038
RF	98.96	85.95	52.73	87.68	86.00	0.4264
DT	80.44	73.51	80.58	91.62	74.00	0.3526
kNN	78.56	67.49	75.16	55.62	67.00	0.2534
SVM	96.26	75.82	92.52	84.20	76.00	0.3596

**Table 5:** Classification accuracy of the proposed autoencoder-SMOTE-Adaboost model with optimal hyperparameters of Adaboost on balance dataset, where N${}_{est}$: number of estimators, l${}_{r}$: learning rate of adaboost, F${}_{e}$: Feature extracted, *Acc${}_{Train}$*: Accuracy on training data, *Acc${}_{Test}$*: Accuracy on test data, Sens: Sensitivity, Spec: Specificity.

N${}_{\mathrm{est}}$	l${}_{r}$	F${}_{e}$	*Acc*${}_{\mathrm{Train}}$ (%)	*Acc*${}_{\mathrm{Test}}$ (%)	Sens. (%)	Spec. (%)
400	0.05	06	90.44	88.29	89.85	82.66
100	0.01	02	88.48	89.54	82.63	91.58
100	0.05	02	88.48	89.54	85.63	78.98
100	0.01	10	87.12	90.00	92.14	83.56
300	0.1	12	89.54	90.16	86.32	91.74
400	0.1	15	92.41	87.58	91.05	86.48
300	0.1	03	89.32	89.54	86.00	90.55
100	0.05	05	88.48	90.00	87.82	95.74
400	0.05	06	92.10	90.23	97.86	98.12
200	0.05	01	88.76	89.54	85.00	81.41
**10**	**0.05**	**06**	**92.10**	**90.23**	**98.00**	**96.65**
50	0.1	04	89.48	90.00	78.36	88.00
50	0.05	07	90.13	86.36	89.05	95.48
200	0.1	06	94.08	86.36	98.00	90.00

**Table 6:** Performance of autoencoder-based predictive models on balanced dataset, where Hyp.: hyperparameters value; F${}_{e}$: feature extracted; *Acc*${}_{Train}$: accuracy on training data; *Acc*${}_{Test}$: accuracy on test data, Sens: sensitivity; Spec: specificity.

Model	Hyp.	F${}_{e}$	*Acc*${}_{\mathrm{Train}}$ (%)	*Acc*${}_{\mathrm{Test}}$ (%)	Sens. (%)	Spec. (%)
AEC * + NB	V = 0.82	14	87.25	87.22	95.56	82.37
AEC + LR	C = 10	15	84.40	87.15	85.35	90.87
AEC + RF	$N_{e}$ = 100	10	100	86.00	52.73	83.45
AEC + DT	$N_{e}$ = 20	18	86.23	88.18	80.58	89.68
AEC + kNN	k = 14	20	100	83.48	79.16	95.32
AEC + SVM	C = 0.5	12	87.15	86.22	92.52	80.28
**AEC + Adaboost**	$N_{e}$ = 10	**06**	**92.10**	**90.23**	**98.00**	**96.65**

AEC *: Autoencoder.

**Table 7:** Classification accuracies comparison with previously proposed methods for dementia prediction.

Study (Year)	Method	Accuracy (%)	Balancing
P.C. Cho & W.H. Chen (2012) [29]	PNNs	83.00	No
P.Gurevich et al. (2017) [26]	SVM	89.00	Yes
D.Stamate et al. (2018) [24]	Gradient Boosting	88.00	Yes
Visser et al. (2019) [25]	XGBoost+ RF	88.00	No
Dallora et al. (2020) [23]	DT	74.50	Yes
M.Karaglani et al. (2020) [27]	RF	84.60	No
E. Ryzhikova et al. (2021) [28]	ANN + SVM	84.00	No
F.A salem et al. (2021) [31]	RF	88.00	Yes
F. G. Gutierrez et al. (2022) [32]	GA	84.00	No
G. Mirzaei,& H. Adeli (2022) [5]	MLP	70.32	No
A. Shahzad et al. (2022) [34]	SVM	71.67	No
**Proposed Model (2022)**	**Autoencoder + SMOTE + Adaboost**	**90.23**	**Yes**

## Data Availability

Data can be available in Supplementary Materials.

## Supplementary Materials

The following supporting information can be downloaded at: https://www.mdpi.com/article/10.3390/life12071097/s1. Table S1: Description of all input variables. Refs. [41,43,44,45,46,47,48,50,67,68] are cited in Supplementary materials.

## Abbreviations

The following abbreviations are used in this manuscript:

PNNs	Probability neural networks
SVM	Support vector machine
MLP	Multilayer perceptron
ML	Machine learning
RF	Random forest
GA	Genetic algorithm
DT	Decision tree
LR	Logistic regression
NB	Naive bayes
kNN	k-nearest neighbors
SMOTE	Synthetic minority oversampling technique
ROC	Receiver operator curve
AUC	Area under the curve
